# Key Signaling Pathways Regulate the Development and Survival of Auditory Hair Cells

**DOI:** 10.1155/2021/5522717

**Published:** 2021-06-11

**Authors:** Yao Liu, Mei Wei, Xiang Mao, Taisheng Chen, Peng Lin, Wei Wang

**Affiliations:** ^1^Department of Otorhinolaryngology Head and Neck Surgery, Tianjin First Central Hospital, 300192 Tianjin, China; ^2^Institute of Otolaryngology of Tianjin, China; ^3^Key Laboratory of Auditory Speech and Balance Medicine, Tianjin, China; ^4^Key Clinical Discipline of Tianjin (Otolaryngology), China; ^5^Otolaryngology Clinical Quality Control Centre, Tianjin, China

## Abstract

The loss of auditory sensory hair cells (HCs) is the most common cause of sensorineural hearing loss (SNHL). As the main sound transmission structure in the cochlea, it is necessary to maintain the normal shape and survival of HCs. In this review, we described and summarized the signaling pathways that regulate the development and survival of auditory HCs in SNHL. The role of the mitogen-activated protein kinase (MAPK), phosphoinositide-3 kinase/protein kinase B (PI3K/Akt), Notch/Wnt/Atoh1, calcium channels, and oxidative stress/reactive oxygen species (ROS) signaling pathways are the most relevant. The molecular interactions of these signaling pathways play an important role in the survival of HCs, which may provide a theoretical basis and possible therapeutic interventions for the treatment of hearing loss.

## 1. Introduction

Globally, hearing loss is the most common sensory disorder, and its severity ranges from mild hearing impairment to total deafness. More than 500 million people are affected by this health problem, and 1 in every 500 newborns worldwide is deaf [1]. Hearing dysfunction may lead to speech retardation, as well as poor social integration and quality of life. Thus far, various causes of hearing loss have been identified and studied, such as aging, ototoxic drugs, genetic mutations of deafness genes, ear or brain tumors, and exposure to loud noises (even for short time) [2, 3]. These causes determine the specific type of hearing loss and the treatment orientation. Hearing loss can be divided into three types according to the location of the lesion: conductive hearing loss (CHL), sensorineural hearing loss (SNHL), and mixed hearing loss. Etiologically, hearing loss can be roughly classified into two categories, including hereditary and nonhereditary [4]. SNHL is an etiologically heterogeneous disorder resulting from numerous genetic and environmental factors [5] and is caused by damage to the body's sound transmission function. Considering that the cochlea is an important sound receiver, analysis of the structure and sound transmission processes of the cochlea form the basis of treatment.

Hair cells (HCs) and spiral ganglion neurons (SGNs) are the main structures of sound transmission in the cochlea [6–11]. The auditory sensory HCs are located in the organ of Corti and include a row of inner hair cells (IHCs), three rows of outer hair cells (OHCs), and supporting cells. Vibrations caused by sound are amplified by the three rows of OHCs and subsequently reach the cochlea. This is turn causes the deflection of stereocilia on the top surface of IHCs, which opens mechanoelectrical transduction channels located at the tip of the stereocilia, and simultaneously leads to excitation of the SGNs [12]. The inner ear and the brain then collaborate to make us feel sound [13]. Hearing loss is caused by the irreversible loss of sensory HCs and degeneration of SGNs [14–19]. Middle ear lesions, noise, trauma, and genetic mutations can cause damage to HCs, which ultimately lead to hearing loss [20–24].

Several signaling cascades are activated following injury to the cochlea. These pathways can be proinflammatory, prodeath, and even prosurvival. The signaling cascades that occur at the cellular and molecular levels are highly complex and intertwined [25]. However, there is relatively little information about the cellular processes that mediate HC damage. As research and treatment in this area are critical, research regarding the cellular molecular pathway mechanisms has attracted considerable attention.

At present, there are an increasing number of studies that have focused on cellular molecular pathways that could represent potential checkpoints in the mechanism of hearing loss and HC damage. As research into the necrobiology of the inner ear progresses, the pathways specific to auditory HC death will become better defined. Among the identified pathways, mitogen-activated protein kinase (MAPK), phosphoinositide-3 kinase/protein kinase B (PI3K/Akt), Notch/Wnt/Atoh1, calcium channel, and oxidative stress/reactive oxygen species (ROS) signaling pathways are the most relevant [26–29]. Importantly, drug interventions in the majority of these processes can achieve benefit.

To summarize the research regarding hearing loss based on cellular molecular pathways in recent years and to guide hearing recovery and the prevention of hearing loss, this paper reviews the current research on the biology of HC damage. The various cellular molecular pathways are discussed systematically. The potential of damage and survival mechanisms as targets for pharmacological intervention to prevent or ameliorate hearing loss is reviewed, and conclusions are drawn in the last section.

## 2. MAPK Signaling

**Figure 1:**
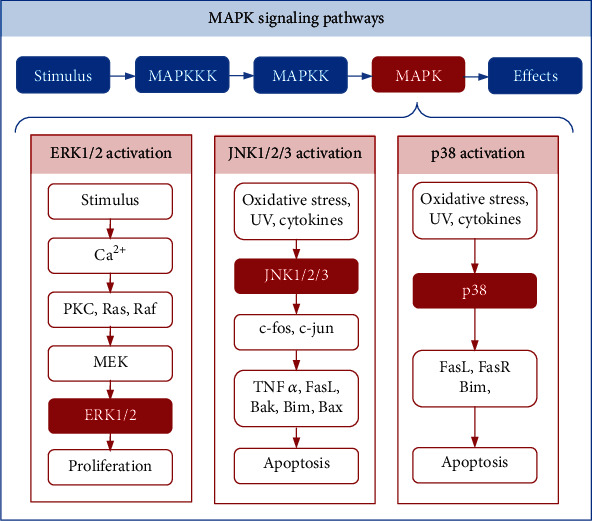
MAPK signaling pathways. There are three tiers of protein kinases that compose each family of MAPKs: MAPKKK, MAPKK, and MAPK. The three main classes of MAPK include ERK1/2, JNK1/2/3, and p38. Stimuli can trigger Ca^2+^, PKC, Ras, Raf, MEK, and ERK1/2 to maintain proliferation and differentiation. Oxidative stress, UV irradiation, and cytokines can trigger JNK and p38 to activate the downstream molecules that promote apoptosis.

Metabolomic and bioinformatic analyses have indicated that MAPK signaling is the major pathway in various types of hearing loss [30, 31]. In the process of evolution, the MAPK cascade reaction has been conserved and consists of three tiers of protein kinases, namely, MAPKKK, MAPKK, and MAPK. This cascade regulates various cellular effects. There are several distinct groups of MAPKs; however, the most extensively studied is extracellular signal regulated kinases (ERK) 1 and 2 (ERK1/2), c-Jun N-terminal kinases (JNK 1, 2, and 3), and p38 kinases [32]. They are activated by various factors; for example, ERK1/2 can be activated by growth factors, and JNK and p38 signaling can be activated by oxidative stress, UV irradiation, hypoxia, and various inflammatory factors (Figure 1).

The MAPK/ERK pathway is reportedly associated with cell proliferation, differentiation, migration, senescence, and apoptosis. It consists of a series of proteins including Ras, Raf, MEK, and ERK. In many cases, ERK1/2 activation is thought to promote cell proliferation and survival [33]. Studies have shown that inhibiting the activation of ERK1/2 in cochlear leads to the loss of OHCs, and gentamicin-induced toxicity is also enhanced [34]. Furthermore, an increasing number of studies have found that ERK1/2 plays an important role in the transformation of supporting cells into HCs. For example, within minutes of a mechanical injury to the cochlear, ERK1/2 signaling is transiently activated in Deiters' and phalangeal cells (supporting cells) but not in HCs [35]. In addition, insulin-like growth factor 1 (IGF-1) was found to activate the MEK/ERK pathway and induce cell cycle promotion of Hensen's and Claudius' cells, both of which are auditory supporting cells that are located lateral to the OHCs of the cochlea. The promotion of this cell cycle in the supporting cells results in the maintenance of the OHC numbers [36]. Another study also showed that forskolin- (FSK-) treated cochlear explants increase the level of cyclic adenosine monophosphate (cAMP) in the auditory supporting cells, thus identifying the MAPK/Raf/ERK pathway as an important downstream signaling pathway in FSK-induced supporting cell proliferation [37]. Therefore, there is substantial evidence indicating that the activation of ERK1/2 in supporting cells can maintain the survival of OHCs.

JNKs, also known as stress-activated protein kinases, phosphorylate the transcription factor, c-Jun. JNK has three isoforms: JNK1, JNK2, and JNK3. The MAPK/JNK signal transduction pathway is activated in response to the exposure of cells to environmental stress and contributes to an apoptotic response by stressed cells that have been damaged by ROS [38]. Studies have shown that aminoglycosides can activate the JNK pathway via ROS, which is an enzyme of the MAPK family signaling pathway, and has been shown to contribute to the apoptosis of damaged cells [38]. In addition, ROS can activate apoptosis signal-regulating kinase-1 (ASK-1), which can phosphorylate and activate mediators of the JNK and p38 pathways of extrinsic programmed cell death [39]. It is well known that both c-fos and c-jun belong to a group of transcription factors called immediate early genes, which can promote apoptosis in response to cellular stress [40]. MAPK/JNK signaling is able to affect both c-fos and c-jun transcription factors, thereby promoting the expression of proapoptotic genes (such as TNF*α*, FasL, Bak, Bim, and Bax) and blocking transcription of antiapoptotic genes (such as Bcl-2) [41–47]. Taken together, the MAPK/JNK signaling pathway is activated by oxidative stress-mediated ROS, and the JNK pathway promotes HC apoptosis by acting on downstream transcription factors.

P38 is another downstream protein of the MAPK pathway and is involved in many cellular processes, including inflammation, cell cycle regulation, and apoptosis [48]. It can be activated by a variety of environmental factors and endogenous stimuli [49]. After p38 activation by stress stimuli, prodeath factors such as Bim, FasL, and FasR are also positively regulated [50–52]. Numerous studies have confirmed that inhibiting the activation of p38 has been associated with protection against aminoglycoside, noise, cisplatin, radiation, and tumor necrosis factor alpha- (TNF*α-*) induced ototoxicity [53–59].

In conclusion, different MAPK kinases have different effects on HCs. In the future, treatments and drugs based on the MAPK pathway may play a significant role in HC regeneration and protection from ototoxic drug–induced hearing loss.

## 3. PI3K/Akt Signaling

The gradual loss of HCs leads to hearing loss, and thus identifying specific signaling pathways that promote HC survival is an effective therapy. Among the identified signaling pathways, PI3K is a powerful candidate for improving the survival of HCs. In recent decades, intensive investigations have been carried out towards PI3K pathway activation for the treatment of aminoglycoside-induced [60–62], sudden sensory neural [63, 64], noise-induced [65, 66], and autosomal-dominant hereditary [67] hearing loss. PI3K plays a key role in various cellular processes, including cell survival, growth, and proliferation. PI3K family members are divided into three classes (class I, II, and III) based on their primary structure and regulatory function. Akt is a major downstream target of PI3K and PTEN and acts as a phosphatase to regulate phosphodiol inositol levels on cell membranes and regulate the PI3K signaling pathway [68]. Akt has serine 473 (p-Akt S473) and threonine 308 (p-Akt T308) phosphorylation sites and serves as a common hub in many antiapoptotic pathways. PI3K/Akt signaling is considered to play an important role in the development of HCs and is involved in the proliferation of auricular precursor cells [69].

Ototoxic drugs are the main environmental factors of hearing loss [70–73]. However, cisplatin and aminoglycoside antibiotics such as kanamycin, gentamicin, or tobramycin are used worldwide, which may lead to the death of sensory HCs in the inner ear via downregulated expression of the PI3K/Akt pathway. Therefore, treatments targeting PI3K/Akt have become an area of intense research interest. Jadali et al. demonstrated that increased PI3K signaling activates Akt, which could directly phosphorylate CHK1 or indirectly increase expression level of pCHK1 levels through DNA damage response proteins, such as ATR. Activation of CHK1 allows supporting cells to repair cisplatin-induced DNA damage [74]. It has also been reported that the PI3K signaling pathway may actively maintain the viability of HCs. Activation of PI3K may be useful in promoting the survival of HCs after aminoglycoside-induced toxicity, and the lack of PI3K signaling could be a cause for congenital hearing loss [60]. To protect auditory HCs from gentamicin-induced apoptotic cell death, pasireotide acts as a novel otoprotective peptide via the PI3K/Akt pathway and activates survival genes, reduces caspase signaling, and increases HC survival [61]. Haake et al. reported that dexamethasone treatment could protect HCs against TNF*α*-induced apoptosis in vitro by activation of PI3K/Akt and NF-*κ*B signaling [65]. Zhang et al. showed that epigallocatechin-3-gallate acts via PI3K/Akt signaling in the cochlea to promote cell growth and neuron differentiation [75].

Sudden sensorineural hearing loss (SSNHL) is a rapid and unexplained SNHL that occurs within 72 hours, with hearing loss ≥ 20 dB in at least 2 adjacent frequencies [76, 77]. By identifying the different expressions of miRNAs in SSNHL patients, it was found that most of the significantly altered miRNAs were abundant in the nervous system, which indicated that putative targeted miRNAs are enriched in the PI3K/Akt, Ras, and MAPK signaling pathways to affect the survival of cochlea HCs [63]. In terms of exhibiting a protective effect on cochlear cells, macrophage migration inhibitory factor (MIF), as a proinflammatory cytokine, can protect cochlear cells from oxygen-glucose deprivation- (OGD-) induced injury by activating the Akt-Nrf2-HO-1 pathway [64]. It is known that insulin-like growth factor 1 (IGF1) plays an important role in the treatment of SNHL [78–80]. IGF1 protects HCs from aminoglycosides by activating the IGF1 receptor and its two main downstream pathways, PI3K/Akt and MEK/ERK, thus leading to the upregulation of the Netrin1-encoding gene (NTN1) expression [81].

Increased PI3K/Akt signaling has a protective effect on hearing loss induced by ototoxicity or adverse environmental factors. Understanding the mechanisms of PI3K/Akt signaling may provide therapeutic ideas for combating hearing loss and identifying new disease strategies.

## 4. Notch/Wnt/Atoh1 Signaling

The human cochlea has about 16,000 sensory HCs, which are necessary for normal hearing [82]. In contrast to nonmammalian vertebrates, most mammalian HCs cannot regenerate, resulting in permanent deafness [83–87]. Cross-species microarrays have identified seven different known signaling pathways: TGF*β*, PAX, Notch, Wnt, NF*κ*B, insulin/IGF1, and AP-1 [88]. Among them, Notch and Wnt signaling pathways are highly complex and conserved that control a variety of cellular events necessary for sensory HCs formation, including cell proliferation and cell fate during cochlear development [89–94]. Simultaneously, Notch and Wnt usually interact to regulate upstream and downstream targets, and Atoh1 is discussed as an important target preferentially [95–100].

Atoh1 is one of the most important transcription factors involved in the development of the inner ear [101]. Previous studies have shown that Atoh1 is both necessary and sufficient for the differentiation, survival, maturation, establishment of auditory function, and long-term survival of HCs [102]. Studies have also shown that Atoh1 is capable of converting the phenotype of mature supporting cells into replacement HCs. Within the critical time window for HC survival, the loss of Atoh1 results in a severe loss of supporting cells and defects in the innervation of the cochlea, causing disruption of the entire auditory sensory epithelium [103]. Another study observed upregulated Atoh1 in supporting cells prior to the significant increase in vivo in the number of HCs without cell division. However, the identification of upstream and downstream targets of Atoh1 may better explain the role of Atoh1 in HC development at the molecular level. For example, the coexpression of EYA1, SIX1, and SOX2 as upstream regulators can effectively activate the HC development program [104]. The downstream target genes POU4F3, BARHL1, and GFI1 can promote Atoh1 to drive HC differentiation [105].

Notch signaling plays a key role in Atoh1 regulation and inner ear development. Atoh1-dependent cell development support and sensory patch patterning are currently believed to be dependent on Notch signaling-mediated lateral inhibition [102]. Cheng et al. demonstrated that HC determination or cell fate support is influenced by Notch signaling [106]. Lee et al. demonstrated that Notch signaling causes inhibition of bHLH proteins (HES1 and HES5) to block the action of Atoh1, which leads to inhibition of HC fate [107]. Therefore, inhibiting the Notch signaling pathway to promote the differentiation of supporting cells into HCs may be a strategy for HC regeneration [97]. The production of new HCs from supporting cells could be increased via inhibition of Notch signaling in the damaged cochlea.

During the development of the cochlea, the Notch signaling pathway interacts with the FGF signaling pathway to control the expression of Atoh1 [107]. Doetzlhofer et al. demonstrated that FGF receptor blockage allows supporting cells to be more responsive to Notch inhibition [108]. Another study demonstrated that FGF and Notch signaling inhibit the proliferation of supporting cells in parallel by inhibiting Wnt signaling [109]. Although FGF signaling inhibits proliferation during regeneration, blocking FGF signaling alone is not enough to enhance proliferation [99, 110].

Wnt/*β*-catenin signaling has been confirmed to be upstream of both Atoh1 and SOX2 during the development of cochlear HCs and retina cells [111, 112]. Wnt/*β*-catenin signaling can promote HC regeneration by increasing cell proliferation and Atoh1 expression. The Wnt pathway also interacts with the Notch pathway. Studies have demonstrated that Wnt activation followed by Notch inhibition significantly promotes the transformation of supporting cells into HCs. Lineage tracing has shown that new HCs, predominantly OHCs, arise from inner pillar and third-row Deiter's cells [113, 114]. In the developing cochlea, Wnt acts on the upstream of Atoh1 to regulate the formation of HCs, while Notch-mediated lateral inhibition prevents supporting cells from adopting the HCs fate by inhibiting the Atoh1 expression [91]. Thus, Wnt may act as a bridge between Notch and Atoh1.

Based on the importance of the Notch/Wnt/Atoh1 pathway, the researchers provide novel therapeutic strategies. Pan et al. reported that genetically engineered Atoh1 knockout mice provide a novel model for establishing critical conditions needed to regenerate viable and functional hair cells with Atoh1 therapy [115]. Mizutari et al. showed that the *γ*-secretase inhibitor LY411575 on the Notch signaling pathway results in the formation of new hair cells in the outer hair cell region and a mild reduction in noise-induced auditory brainstem response (ABR) threshold shifts; although, the mice were still functionally deaf [116]. In addition, the in vitro hair cell that yields from Lgr5-positive cells isolated from neonatal mice and grown as organoids can be further improved by treatment with the Wnt activator CHIR99021 (CHIR) and the histone deacetylase (HDAC) inhibitor valproic acid [117, 118].

**Figure 2:**
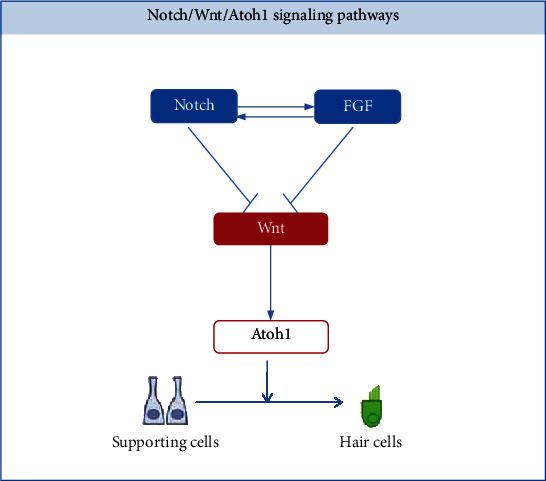
Notch/Wnt/Atoh1 signaling pathways. The FGF signaling pathway interacts with the Notch signaling pathway to inhibit the activation of Wnt. Wnt acts on the upstream of Atoh1 to regulate supporting cells to transdifferentiate into hair cells.

In summary, Notch interacts with the FGF to inhibit the expression of Wnt and further suppress the downstream molecule Atoh1, thereby preventing the differentiation of supporting cells into HCs (Figure 2) [97, 115, 119]. When targeting individual Notch or Wnt signaling in the cochlea, only a modest HC regenerative response (or no response) can be observed [116, 120]. Considering the synergistic role of these signaling pathways in regulating cochlear development, targeting multiple pathways may be a more promising HC regeneration strategy.

## 5. Calcium Channel

Acquired hearing loss, including noise-induced hearing loss, age-related deafness, and ototoxicity-induced hearing loss, has complex mechanisms for each disease. Despite the complexity of these mechanisms, numerous studies have shown that apoptosis of inner ear structures is a common theme among many types of acquired hearing loss [121–123]. Calcium is one of the important cofactors involved in the degradation enzyme of apoptosis, and the interaction between calcium and apoptosis is becoming increasingly obvious [124]. Homeostatic control of calcium ions (Ca^2+^) is critical for cell survival. On this basis, transport channels, ligand-gated channels, and voltage-gated calcium channels (VGCCs) are located on the plasma membrane of HCs and facilitate Ca2^+^ entry into HCs [125]. Also, within the inner ear, L- and T-type calcium channels of VGCCs are believed to contribute to calcium availability during apoptosis. Among them, the family of T-type calcium channels have three members (Cav3.1, Cav3.2, and Cav3.3), based on their respective main pore-forming alpha subunits: *α*1G, *α*1H, and *α*1I [126]. In the T-type calcium channels, Cav3.2 is the most significantly expressed T-type channel entity in the cochlea and auditory brainstem. The results have shown that Cav3.2 VGCCs are of great functional importance for spatiotemporal auditory processing in different regions of the auditory system [127].

Therapeutic agents aimed at preventing apoptosis are a concept central to many therapies that target acquired hearing loss. Research is currently underway to evaluate potential therapeutic targets within the peripheral auditory system and apoptotic pathways. Numerous studies have shown that calcium channel blockers (CCBs) can effectively prevent damage to cochlear cells. More recent in vivo research has provided evidence to suggest that systemic and intratympanic (direct) application of CCBs can prevent hearing loss in cisplatin- and noise-induced ototoxic models [128]. For example, the T-type calcium channel blocker flunarizine can significantly inhibit cisplatin-induced apoptosis; however, this is not mediated by the modulation of intracellular calcium levels. It can inhibit lipid peroxidation and mitochondrial permeability transition in cisplatin-treated cells [129]. Additionally, trimethadione and ethosuximide are two T-type calcium blockers which are antiepileptics approved by the Food and Drug Administration [126, 130]. Both of these drugs can significantly slow age-related auditory brainstem response (ABR) threshold shifts in mice and reduce noise-induced hearing loss when applied prior to noise exposure, most likely through effects on the *α*1H T-type calcium channel subunit comprising one or more Cav3 calcium channel types in the cochlea [130]. In addition, aminoglycosides such as gentamicin could trigger a Ca^2+^ influx that activates proapoptotic signaling cascades in HCs. There is evidence that the Ca^2+^-sensitive neuropeptide, somatostatin (SST), can antagonize aminoglycoside-induced cell death. SST analogs have the same effect [131].

In view of the apoptotic effect of calcium channels in cochlea HCs, these channels may become a potential pharmacological target for clinical interventions in the future. Pharmacological inhibition of CCBs may represent a promising approach to the treatment of auditory impairment of various etiologies.

## 6. Oxidative Stress and ROS Signaling

ROS-induced oxidative stress has been reported to play a key role in several systems and in cochlear damage [132–136]. Numerous studies have demonstrated that the accumulation of ROS and subsequent apoptosis induction promote several major types of SNHL, including noise-induced hearing loss, drug-induced hearing loss, and age-related hearing loss [137–142]. ROS are considered to be toxic products of cell metabolism and are signaling molecules that regulate a variety of physiological processes. ROS, including superoxide anions, hydroxyl radicals, hydrogen peroxide (H_2_O_2_), and singlet oxygen, are mainly generated by the mitochondria in most mammalian cells [143, 144]. In both physiological and pathological conditions, ROS-induced oxidative stress can induce apoptosis via both the extrinsic cell death receptor pathway and the intrinsic mitochondrial cell death pathway. Following a stimulus, mitochondrial aerobic respiration increases, and a large amount of ROS cannot be effectively neutralized. ROS-induced superoxide and lipid peroxidation production can lead to apoptosis, and vasoactive lipid peroxidation products reduce cochlear blood flow and further enhance the production of ROS. Furthermore, the production of ROS in the cochlea will promote the production of proinflammatory cytokines, which will cause further damage [145]. This eventually leads to the death of OHCs (predominantly) and IHCs through either apoptosis or necrosis [146, 147].

Many drugs, including cisplatin, gentamicin, and neomycin, are ototoxic and may cause irreversible apoptosis of cochlear HCs. These can increase the levels of ROS, which ultimately leads to cell injury and mitochondrial dysfunction. As for the important role of ROS in cell apoptosis, considerable research has been focused on inhibiting ROS production. For example, Guo et al. reported that FSK significantly reduced cisplatin-induced ototoxicity in both HEI-OC1 cells and cochlear explant cultures by inhibiting the mitochondrial apoptotic pathway and ROS production [107]. Shin et al. also demonstrated that KR-22332 exhibits similar mechanisms to prevent cisplatin-induced ototoxicity [148]. Quan et al. demonstrated that adjudin can regulate ROS production in cochlear cells and inhibit gentamicin-induced production of ROS and apoptotic cells [149].

Increased oxidative stress and ROS play an important role in the initiation and progression of hearing loss induced by diverse ototoxic agents or adverse health conditions. Understanding the mechanisms of oxidative stress and ROS signaling may provide therapeutic options for combating hearing loss and identifying new treatment strategies.

## 7. Conclusion

**Figure 3:**
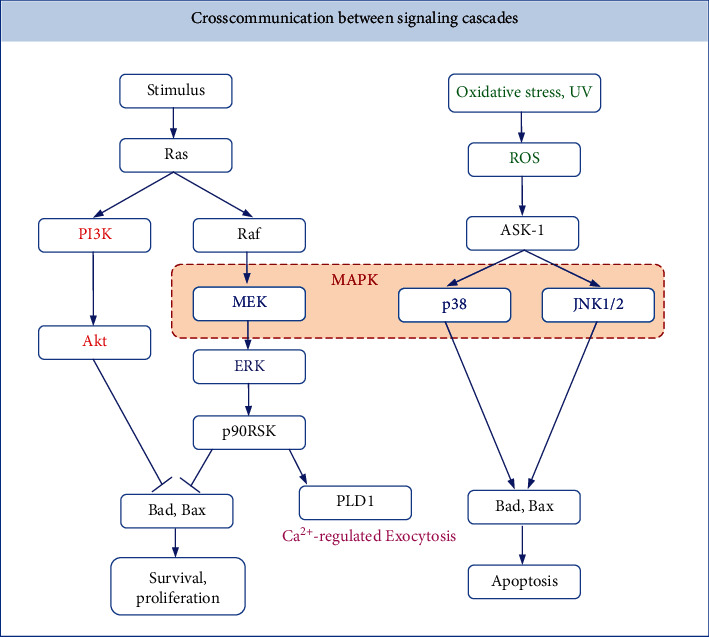
Crosscommunication between signaling cascades. Both PI3K/Akt and MEK/ERK signaling pathways can inhibit the expression of proapoptotic proteins Bad and Bax and promote the survival and proliferation of hair cells. ROS can act on the p38 and JNK pathways through ASK-1 and promote the expression of proapoptotic proteins Bad and Bax to induce the apoptosis of hair cells.

Numerous types of inner ear damage can cause harm to auditory HCs and ultimately result in hearing loss. Multiple signaling pathways are involved in ototoxicity, noise, aging, and traumatic stress events. The most well studied molecular mechanisms behind cell death in auditory HCs are the MAPK, PI3K/Akt, Notch/Wnt/Atoh1, calcium channel, and oxidative stress/ROS signaling pathways. In this review, we provide a schematic showing how the listed pathways interact within the hair cells [71, 150–152] (Figure 3). Although their effects on cochlear HC proliferation and survival have been studied extensively, there are likely many levels of crosscommunication between signaling cascades that are still undiscovered. Research in this field is becoming increasingly prevalent, as is research into the mechanisms of regulated development and survival of auditory HCs. A number of otoprotective drug therapies target different levels along these signaling pathways to promote auditory HC viability and hearing protection.
